# Randomised trial of population‐based *BRCA* testing in Ashkenazi Jews: long‐term secondary lifestyle behavioural outcomes

**DOI:** 10.1111/1471-0528.17253

**Published:** 2022-07-13

**Authors:** Matthew Burnell, Faiza Gaba, Monika Sobocan, Rakshit Desai, Saskia Sanderson, Kelly Loggenberg, Sue Gessler, Lucy Side, Angela F. Brady, Huw Dorkins, Yvonne Wallis, Chris Jacobs, Rosa Legood, Uziel Beller, Ian Tomlinson, Jane Wardle, Usha Menon, Ian Jacobs, Ranjit Manchanda

**Affiliations:** ^1^ MRC Clinical Trials Unit at UCL, Institute of Clinical Trials and Methodology University College London London UK; ^2^ Wolfson Institute of Population Health, Barts CRUK Cancer Centre Queen Mary University of London London UK; ^3^ Department of Gynaecological Oncology Barts Health NH Trust London UK; ^4^ Behavioural Sciences Unit Department Epidemiology and Public Health University College London London UK; ^5^ Department Clinical Genetics North East Thames Regional Genetics Unit Great Ormond Street Hospital London UK; ^6^ Department of Gynaecological Oncology Institute for Women's Health University College London London UK; ^7^ University Hospital Southampton NHS Foundation Trust Southampton UK; ^8^ Department Clinical Genetics North West Thames Regional Genetics Unit Northwick Park Hospital London UK; ^9^ St Peter's College University of Oxford Oxford UK; ^10^ West Midlands Regional Genetics Laboratory Birmingham Women's NHS Foundation Trust Birmingham UK; ^11^ Depatment Clinical Genetics West Midlands Regional Genetics Service Birmingham Women's NHS Foundation Trust Birmingham UK; ^12^ Depatment Clinical Genetics Guy's Hospital London UK; ^13^ University of Technology Sydney Sydney New South Wales Australia; ^14^ Department of Health Services Research and Policy London School of Hygiene & Tropical Medicine London UK; ^15^ Department of Gynaecology Shaare Zedek Medical Center Jerusalem Israel; ^16^ Institute of Cancer and Genomic Sciences University of Birmingham Birmingham UK; ^17^ University of New South Wales Sydney New South Wales Australia; ^18^ Department of Gynaecology All India Institute of Medical Sciences New Delhi India

**Keywords:** Ashkenazi Jews, *BRCA1/BRCA2*, cancer risk, genetic testing, lifestyle, population testing

## Abstract

**Objective:**

Ashkenazi‐Jewish (AJ) population‐based *BRCA* testing is acceptable, cost‐effective and amplifies primary prevention for breast & ovarian cancer. However, data describing lifestyle impact are lacking. We report long‐term results of population‐based *BRCA* testing on lifestyle behaviour and cancer risk perception.

**Design:**

Two‐arm randomised controlled trials (ISRCTN73338115, GCaPPS): (a) population‐screening (PS); (b) family history (FH)/clinical criteria testing.

**Setting:**

North London AJ‐population.

**Population/Sample:**

AJ women/men >18 years. Exclusions: prior *BRCA* testing or first‐degree relatives of *BRCA*‐carriers.

**Methods:**

Participants were recruited through self‐referral. All participants received informed pre‐test genetic counselling. The intervention included genetic testing for three AJ *BRCA*‐mutations: 185delAG(c.68_69delAG), 5382insC(c.5266dupC) and 6174delT(c.5946delT). This was undertaken for all participants in the PS arm and participants fulfilling FH/clinical criteria in the FH arm. Patients filled out customised/validated questionnaires at baseline/1‐year/2‐year/3‐year follow‐ups. Generalised linear‐mixed models adjusted for covariates and appropriate contrast tests were used for between‐group/within‐group analysis of lifestyle and behavioural outcomes along with evaluating factors associated with these outcomes. Outcomes are adjusted for multiple testing (Bonferroni method), with *P* < 0.0039 considered significant.

**Outcome measures:**

Lifestyle/behavioural outcomes at baseline/1‐year/2‐year/3‐year follow‐ups.

**Results:**

1034 participants were randomised to PS (*n* = 530) or FH (*n* = 504) arms. No significant difference was identified between PS‐ and FH‐based *BRCA* testing approaches in terms of dietary fruit/vegetable/meat consumption, vitamin intake, alcohol quantity/ frequency, smoking behaviour (frequency/cessation), physical activity/exercise or routine breast mammogram screening behaviour, with outcomes not affected by *BRCA* test result. Cancer risk perception decreased with time following *BRCA* testing, with no difference between FH/PS approaches, and the perception of risk was lowest in *BRCA*‐negative participants. Men consumed fewer fruits/vegetables/vitamins and more meat/alcohol than women (*P* < 0.001).

**Conclusion:**

Population‐based and FH‐based AJ *BRCA* testing have similar long‐term lifestyle impacts on smoking, alcohol, dietary fruit/vegetable/meat/vitamin, exercise, breast screening participation and reduced cancer risk perception.

## Tweetable Abstract

Jewish Pop *BRCA* testing increases vitamin use and has similar lifestyle impact as FH testing on diet, exercise, alcohol, breast screening.

## 1 INTRODUCTION

Population‐based *BRCA* testing in the Ashkenazi Jewish (AJ) population has been shown to be feasible, acceptable, effective, does not adversely affect long‐term psychological well‐being or quality‐of‐life, and is cost‐effective, projected to save both lives and money.^1^, ^2^, ^3^, ^4^, ^5^, ^6^, ^7^ It provides the opportunity for maximising identification of *BRCA*‐carriers for primary prevention of breast cancer (BC) and ovarian cancer (OC). *BRCA* testing provides information on cancer risk, which in turn can influence lifestyle habits such as diet, alcohol, smoking and physical activity. While it is hoped that genetic test results will lead to beneficial lifestyle effects to mitigate risk, behaviour change is not easy. Presenting risk information does not necessarily lead to strong sustainable changes in health behaviour.^8^ Data on the impact of *BRCA* testing on lifestyle behavioural factors are limited. A small number of studies with short‐term follow‐up have evaluated this in high‐risk populations and shown inconsistent results.^9^, ^10^, ^11^ Data on lifestyle impact of *BRCA* testing through population‐based ascertainment are lacking. It is also unknown whether *BRCA* testing on a large scale using a population‐based approach (given that the huge majority of individuals will test negative) could lead to false reassurance and have a detrimental impact on lifestyle behaviours such as smoking, alcohol consumption, diet, physical exercise and routine mammography screening. Lifestyle factors such as nutrition, physical activity, smoking and alcohol are important because they can affect chronic diseases such as heart disease, stroke, diabetes, lung disease and cancer.^12^, ^13^, ^14^, ^15^, ^16^ While they may or may not have a direct impact on *BRCA*‐associated BC/OC‐risk in PV‐carriers (substantive data on this are lacking), they are of importance for their association with chronic disease, particularly in the majority testing negative. Chronic disease‐related treatment costs are a massive drain on all health systems. These account for 64% outpatient/70% inpatient workload, and 70% of total UK healthcare expenditure^17^ as well as 90% Medicare expenditure, contributing to 2.7 million deaths annually in the USA.^18^

Data from population cohort studies show that BC risk perception in non‐carriers decreases over a year and accuracy of risk perception improves with pre‐test counselling in high‐risk women.^19^ However, randomised controlled trial (RCT) data regarding associated changes in cancer risk perception (between population‐based and criteria/family history (FH)‐based testing) are lacking. The Jewish population is the first population for whom population‐based genetic testing for cancer prevention is now being implemented. To facilitate informed decision making for population testing, it is important also to evaluate and understand any potential impact of *BRCA* testing on general lifestyle factors. We have previously reported on primary outcomes of psychosocial well‐being and quality‐of‐life following *BRCA* testing.^3^, ^7^ We now report on long‐term (up to 3 years) results of lifestyle behaviour‐related secondary outcomes of: (a) diet (b) alcohol intake (c) smoking, (d) exercise, as well as (e) cancer risk perception, (f) routine breast screening uptake following population‐based *BRCA* testing (compared with criteria/FH testing) in the Genetic Cancer Prediction through Population Screening (GCaPPS) trial (ISRCTN73338115). We also evaluate the association of demographic and epidemiological factors with these outcomes.

## 2 METHODS

### 2.1 Design

North London‐based self‐referred consenting AJ individuals >18 years were randomised (1:1) using a computer‐generated random‐number algorithm to either population‐screening (PS arm) or FH‐based testing (FH arm). All underwent pre‐test genetic counselling.^3^ Counsellors were blinded to group allocation. All participants in the PS arm and only those fulfilling standard FH‐based criteria in the FH arm underwent genetic testing for the three Jewish *BRCA* pathogenic variants (PVs), called founder mutations: 185delAG(c.68_69delAG), 5382insC(c.5266dupC) and 6174delT(c.5946delT). Individuals with previous *BRCA* testing, and first‐degree relatives (FDR) of *BRCA*‐carriers were excluded. Individuals with PVs received post‐test counselling. These details have been described in earlier reports on psychological (primary) outcomes^3^, ^7^ (90% power for detecting a HADS‐score difference = 1.2 between study arms; SD = 5.9; α = 0.05). We now report on long‐term (up to 3‐years) secondary outcomes of lifestyle behaviour: (a) diet (b) alcohol intake, (c) smoking, (d) exercise, as well as (e) cancer risk perception and (f) routine breast screening uptake. We report between‐group (FH and PS arms) differences adjusted for any baseline difference collectively across 1, 2 and 3 years. We also assessed time effects for each group (within‐FH group and within‐PS group) from baseline over 1, 2 and 3 years. We explored the association of gender, income, BRCA status and FH of cancer and age on these outcomes and adjusted for them in the analysis. Customised questionnaires were used to collect socio‐demographic, FH data and outcome data. Data were collected at baseline and then annually for 3 years.

Standardised items obtained from the UK National Diet & Nutrition Survey (NDNS); Health Survey England (HSE), Harvard Center for Cancer‐Prevention's Harvard Cancer Risk Index (HCRI), and CDC Behavioral Risk‐Factor Surveillance System (BRFSS) were used to assess these outcomes. The NDNS is a rolling programme collecting information on food consumption, nutrient intake and nutritional status of the UK general population funded by Public Health England (PHE) and the UK Food Standards Agency (FSA). HSE is an annual survey monitoring changes in the health and lifestyles of people across the UK. Cancer‐related food consumption was measured with three items adapted from the NDNS: ‘Please indicate how often, on average, you eat each of the following foods: red meat, fruit, vegetables'; with seven response options (ranging from ‘Never’ to ‘More than once a day’), which were categorised for analysis to: <once/week, ≥ once/week and ≥ once/day. Vitamin consumption was assessed with an item adapted from NDNS: ‘Are you taking any vitamin supplements?’ (no/yes). Alcohol consumption was assessed with one item adapted from the NDNS: ‘How often have you had an alcoholic drink during the last 12 months?’ (response options: ≤ twice/year; every 2 months; ≤ twice/month; 1–2 days/week; ≥3 days/week), plus one item adapted from the Harvard Center for Cancer Prevention ‘Your Disease Risk’ website, which is based on the HCRI (Colditz,2000; https://siteman.wustl.edu/prevention/ydr/): How many servings of alcohol do you have on a typical day? (0/1/2/3 or more).

Physical activity was assessed with one item from the CDC‐BRFSS (http://www.cdc.gov/brfss/questionnaires/pdf‐ques/2006brfss.pdf): ‘During the past month, other than your regular job, did you participate in any physical activities or exercises such as running/golf/gardening/walking for exercise?’ (yes/no); one item from the HCRI: ‘Do you walk (or do other moderate activity) for at least 30 minutes most days, or at least 3 hours/week?’ (yes/no), and one item adapted from the HSE: ‘Thinking about your job in general, would you say that you are very/fairly/not very/not at all physically active’.

Smoking was assessed with items adapted from the CDC‐BRFSS: ‘Do you now smoke cigarettes every day, some days, or not at all?’ and ‘During the past 12 months, have you stopped smoking for 1 day or longer because you quit or were trying to quit smoking?’ (yes/no).

Comparative cancer risk was assessed with (a) ‘Compared with other people of your age and sex, do you think your chances of getting cancer at some point in your life are: much lower, lower, about the same, higher, much higher?’ (Sutton, 1994); and (b) ‘On a scale from 0 to 100, where 0 is no chance at all and 100 is absolutely certain, what do you think are the chances that you will get cancer sometime during your lifetime?’ (Smith, 2004). Use of a mammogram was assessed with items adapted from the CDC‐BRFSS: Have you ever had a mammogram?’ [yes/no/not sure]; ‘If yes, how long has it been since you had your last mammogram?’ [<1 year, >1 year, not sure].

### 2.2 Statistical analysis

The responses to the lifestyle questions at ‘baseline’, 1 year, 2 years and 3 years were analysed using generalised linear mixed models, where a random intercept term represented the unexplained heterogeneity corresponding to each subject. Specifically, a logistic mixed‐model was fitted for the binary items and an ordinal logistic regression model fitted for ordered multiple response items. Questions on breast screening (mammogram) were re‐formulated to be a binary response variable that asked whether the subject had the screening/mammogram within the last 12 months and responses were analysed using logistic mixed‐models. The analysis of mammogram outcomes was restricted to women >50 years. Each time‐point was included as a fixed‐effect and interacted with the group term (‘family history’ or ‘population screening’), resulting in all eight group and time mean values being freely estimable. In addition, the model was adjusted for gender (men versus women), *BRCA* founder‐mutation status (positive or negative or unknown), marital status (married/cohabiting versus widowed/divorced/single), income (<£10,000, £10,000 to <£20,000, £20,000 to <£30,000, £30,000 to <£40,000, £40,000 to <£50,000, and >£50,000), education (degree level/above versus no formal qualification/GCSE/O‐level/CSE/NVQ1/NVQ2/A‐level education), family history (low‐risk versus high‐risk) and age.

A linear combination of the relevant time and group parameters for each model was used to estimate the accumulated difference between groups over time (years 1, 2 and 3) in the logit scale, accounting for any baseline difference. A similar linear combination of group‐specific parameters for each model was calculated to estimate the total change from baseline (over years 1, 2 and 3) for each group in the logit = log(odds) scale. This is the cumulative difference between groups over years 1, 2 and 3 (accounting for the baseline difference). As 13 variables were analysed, results were adjusted for multiple testing. We were conservative and used a Bonferroni correction, with *P‐*values <0.0039 (0.05/13) considered to be significant. Potential group differences over the four time‐points were also explored visually to help interpret the model parameters for group when interacted with time. To visualise any trends, the STATA margin command was used to make predictions over the sample for each of the eight group‐by‐time interactions. Specifically, for the binary items, the marginal prediction was the population averaged (that is, after integrating over the random effect for subject) probability of a positive response. For the ordered items, the same predictions were made for each of the possible outcome categories. These marginal predictions with their confidence intervals were then plotted. Statistical analyses used STATA‐17.0 (StataCorp LP).

2.2.1 | Core outcome sets (COS)

There are no COS for population *BRCA* testing at present.

2.2.2 | Patient & public involvement (PPI)

The GCaPPS trial was preceded by a year‐long extensive community engagement process with all denominations in the Jewish community, involving a broad range of stakeholders. This included representatives from the Liberal, Reform, Masorti/Conservative, Orthodox and Unaffiliated community denominations. We engaged with a range of stakeholders including religious leaders, Rabbis, Boards of Deputies, Jewish charities, Jewish Medical Association, cancer charities and patient support groups. This exercise enabled exchanges of ideas and understanding of underlying concerns related to *BRCA* testing and the research. It provided community inputs into study protocol development, communication strategy, development of participant/patient‐facing materials, study conduct/delivery. There was PPI/public representation on trial steering and management committees. The extensive PPI exercise was instrumental in generating support, awareness and successful completion of the study.

## 3 RESULTS

In all, 1034 AJ participants were recruited: 530 were randomised to the PS arm and 504 to the FH arm; 691 women and 343 men consented to *BRCA* testing and were randomised. In the PS arm, 530 participants and in the FH arm 66 participants underwent *BRCA* testing. The consort flow‐chart is given in Figure S1. Baseline characteristics of the two study arms have been reported earlier and were comparable.^3^, ^7^ The questionnaire response rates at baseline were 99%, at 1 year 77–80%, at 2 years 71–72%, and at 3 years 64–71% (Figure S1).

**FIGURE 1:**
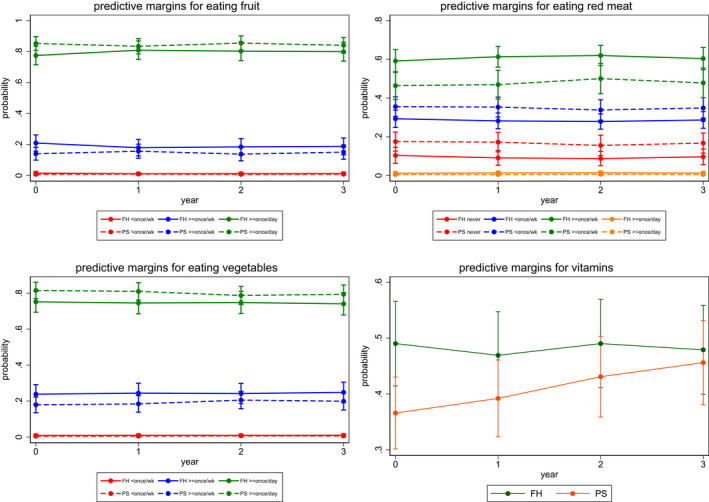
Dietary fruit, vegetable, meat and vitamin consumption by group over time. FH, family history; PS, population screening.

**FIGURE 2:**
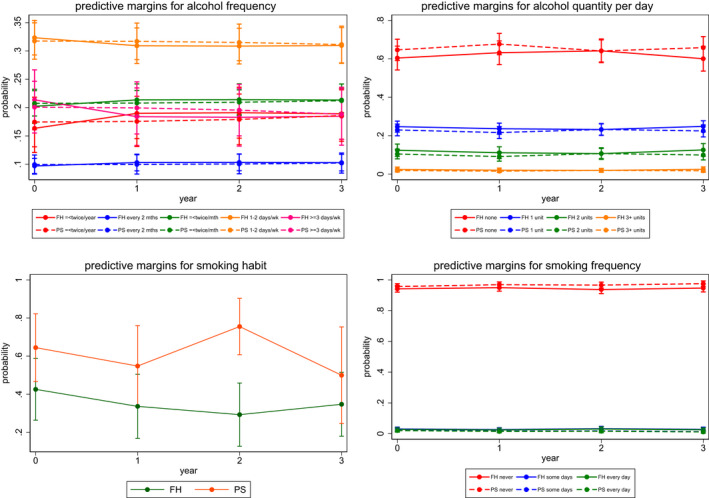
Alcohol consumption and smoking by group over time. FH, family history, PS, population screening.

**FIGURE 3:**
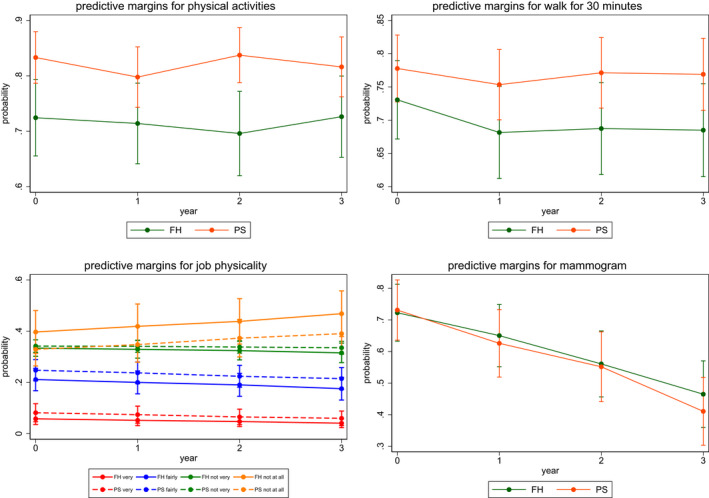
Change in physical activity and breast screening behaviour by group over time. FH, family history; PS, population screening. Physical activities: physical activities or exercises such as running/golf/gardening/walking for exercise?’ Walk for 30 minutes: reflects walking or other moderate activity for at‐least 30 minutes on most days or at least 3 hours/week. Job physicality: level of physical activity in a person's job—‘very/fairly/not very/not at all physically active’.

Estimates of the accumulated difference in outcomes between FH and PS groups over time (years 1, 2 and 3) are given in Table 1. Figures 1, 2, 3 provide a visual interpretation of outcome variables by group over time. Additionally, the effect sizes of the within‐group difference of outcome variables with confidence intervals are given in Table 1. We found no difference in dietary fruit, vegetable or meat consumption between FH‐ and population‐testing approaches over time or any significant change in levels of intake over time (Figure 1; Table 1). There was a significant increase in vitamin supplement intake (coefficient = 1.778, 95% CI 0.69–2.86; *P* = 0.001) within the population‐testing arm alone. However, this difference (coefficient = 2.075, 95% CI 0.52–3.63) was not statistically significantly different (*P* = 0.03; below the *P* = 0.0039 threshold of significance) compared with the intake seen with FH/criteria‐based testing over time (Figure 1; Table 1). There was no difference in the quantity or frequency of alcohol consumed between PS arm and FH arm participants over time (Table 1; Figure 2). We did not find any change in smoking behaviour (either smoking frequency or smoking cessation) between the two *BRCA*‐testing approaches or associated with smoking behaviour overall with time (Table 1; Figure 2). There was no significant difference observed with respect to physical activity related to regular exercise, walking/moderate activity or job‐related activity between groups over time (Figure 3; Table 1). However, overall job‐related activity decreased with time in both FH (coefficient = 1.204, 95% CI 0.21–2.19, *P* = 0.017) and PS (coefficient = 1.204, 95% CI

**TABLE 1:** Outcomes for between‐group difference and within‐group differences over time

	between FH and PS groups			Within FH Group			Within PS group		
Outcome variable	Coeff	95% CI	*P*‐value	Coeff	95% CI	*P*‐value	Coeff	95% CI	*P*‐value
Diet: fruit	−1.596	−3.42 to 0.23	0.087	1.164	−0.11 to 2.44	0.073	−0.431	−1.74 to 0.88	0.518
Diet: red meat	−0.274	−1.79 to 1.25	0.724	0.925	−0.15 to 2.01	0.093	0.651	−0.42 to 1.72	0.234
Diet: vegetable	−0.544	−2.38 to 1.29	0.560	−0.294	−1.58 to 0.99	0.655	−0.838	−2.14 to 0.47	0.208
Vitamins	2.075	0.52–3.63	0.009	−0.297	−1.40 to 0.81	0.597	1.778	0.69–2.86	0.001
Alcohol: quantity	0.311	−1.09 to 1.71	0.664	−0.796	−1.80 to 0.21	0.122	−0.485	−1.46 to 0.49	0.330
Alcohol: frequency	1.422	0.22–2.62	0.02	−1.809	−2.67 to −0.95	<0.001	−0.386	−1.23 to 0.46	0.370
Smoking stopping	−1.038	−4.52 to 2.44	0.559	1.678	−0.70 to 4.06	0.167	0.640	−1.91 to 3.19	0.623
Smoking: frequency	−1.116	−2.92 to 0.69	0.225	−0.127	−1.18 to 0.92	0.813	−1.243	−2.71 to 0.23	0.098
Physical activity: exercise	−0.215	−1.77 to 1.34	0.787	−0.332	−1.42 to 0.76	0.550	−0.547	−1.67 to 0.57	0.339
Physical Activity: walking	1.140	−0.53 to 2.81	0.181	−1.672	−2.88 to −0.46	0.007	−0.532	−1.69 to 0.63	0.368
Physical activity: Job	0.001	−1.35 to 1.35	0.999	1.204	0.21–2.19	0.017	1.204	0.27–2.14	0.011
Cancer risk perception	−1.173	−2.39 to 0.04	0.059	−1.732	−2.60 to −0.87	<0.0001	−2.905	−3.78 to −2.03	<0.0001
Mammogram	−0.650	−2.51 to 1.21	0.494	−2.85	−4.13 to −1.56	<0.0001	−3.50	−4.87 to −2.12	<0.0001

*Note*: Between‐FH and ‐PS groups estimates the accumulated difference (in the outcome variable) between FH and PS groups over time (years 1, 2 and 3), accounting for any baseline difference. Within‐FH group estimates the total change in the outcome variable from baseline (over years 1, 2 and 3) for the FH group. Within‐PS group estimates the total change in the outcome variable from baseline (over years 1, 2 and 3) for the PS group.

95% CI, 95% confidence interval; Coeff, coefficient; FH, family history; PS, population screening.

0.27–2.14; *P* = 0.011) groups (Table 1). We did not find any difference in uptake of routine mammogram screening between PS and FH approaches to *BRCA* testing with time in women >50 years age, although uptake decreased with time (Figure 3; Table 1). In addition, the lack of difference observed between *BRCA* testing approaches in terms of dietary fruit/vegetable/meat consumption, vitamin intake, alcohol quantity/frequency, physical activity/exercise or routine breast mammogram screening behaviour was not affected by a positive or negative *BRCA* test result. Overall, there was a significant decrease in perceived cancer risk over 3 years following both FH‐based (*P <* 0.001) and PS‐based (*P* < 0.001) *BRCA* testing, but no difference was observed between FH or PS approaches to *BRCA* testing (*P* = 0.06) (Table 1). However, *BRCA*‐negative indivuduals perceived their cancer risks to be lower than those testing positive or those of unknown *BRCA*‐status.

The outputs of the generalised linear mixed‐models showing the association of covariates with the different outcomes are given in Table S2. Modelling suggested that men eat fewer fruits (coefficient −1.805, 95% CI −2.377 to −1.233; *P <* 0.0001), vegetables (coefficient −2.262, 95% CI −2.848 to −1.677; *P <* 0.0001) and vitamins (coefficient −1.597, 95% CI −2.132 to −1.062; *P <* 0.0001) but consume more meat (coefficient 1.844, 95% CI 1.149–2.538; *P <* 0.0001) and alcohol in greater quantities (coefficient 2.483, 95% CI 1.884–3.080; *P <* 0.0001) and more frequently (coefficient 3.064, 95% CI 2.30–3.83; *P <* 0.0001) than women do (Table S1). Men are more physically active in their jobs (*P* = 0.01) and perceive their cancer risk to be lower compared with women (*P <* 0.0001) (Table S1). Higher levels of education were associated with greater fruit (coefficient 0.879, 95% CI 0.307–1.451; *P* = 0.003) or vegetable intake (coefficient 1.074, 95% CI 0.502–1.647; *P <* 0.0001) and more physically active jobs (*P* = 0.002) (Table S1). Participants lacking a strong FH perceived their cancer risk to be lower (coefficient −0.607, 95% CI –1.075 to –0.139; *P* *<* 0.0001). *BRCA‐*carriers perceived their cancer risk to be higher than non‐carriers. However, the effect size was greater for those testing negative (coefficient −3.227, *P* < 0.0001) than for those who did not receive a test result (coefficient −2.308, *P* = 0.014). Older participants consumed greater amounts of fruits (coefficient 0.064, 95% CI 0.043–0.084; *P <* 0.0001), vegetables, vitamins (coefficient 0.038, 95% CI 0.020–0.056; *P <* 0.0001) and alcohol (coefficient 0.037, 95% CI 0.017–0.057; *P <* 0.0005) but less meat (coefficient −0.024, 95% CI −0.047 to −0.001; *P* = 0.043) and perceived their cancer risks to be lower (coefficient −0.040, 95% CI −0.056 to −0.023; *P <* 0.0001). In women >50 years of age, mammogram uptake decreased with age (coefficient −0.069, 95% CI –0.099 to –0.039; *P <* 0.001) but there was no difference in mammogram screening uptake between the groups.

## 4 DISCUSSION

### 4.1 Main findings

To the best of our knowledge this is the only RCT study on lifestyle impact comparing population‐based *BRCA* testing with FH‐based testing. Our findings are reassuring, as they do not show a significant difference or any detrimental impact on overall lifestyle factors, such as dietary fruit, vegetable or meat intake, alcohol, smoking or physical activity and exercise, with population‐based *BRCA* testing compared with the standard FH‐based testing. We found an increase in vitamin intake within the population‐based *BRCA* testing arm. Men eat fewer fruits, vegetables, vitamins and consume more alcohol and meat than women. Fruit, vegetable, alcohol and vitamin consumption increased, whereas meat intake decreased with age. Non‐carriers perceived themselves at lower risk than *BRCA‐*carriers and overall cancer risk perception decreased with time, but there was no difference between the PS and FH approaches. Routine mammogram screening uptake declined with time for average‐risk women over 50 years, but there was no difference between the FH and PS arms.

### 4.2 Strengths and weaknesses

Our study has a number of advantages, including population‐based ascertainment, inclusion of men and women, randomised design, adequate questionnaire response rate and long‐term follow‐up. Its limitations include lack of qualitative data and inferences from *BRCA* testing in the AJ‐population not being directly generalisable to the non‐Jewish general population.

### 4.3 Interpretation

Although genetic testing can be used to facilitate healthy lifestyle behaviours, a clear cause‐and‐effect association has yet to be demonstrated.^20^ Lifestyle factors such as diet (reduced vegetable/fruit), reduced physical activity/exercise, smoking and alcohol can also increase risk of chronic diseases such as heart disease, stroke, diabetes, lung disease and cancer.^12^, ^13^, ^14^, ^15^, ^16^ That these were not negatively affected by population‐based *BRCA* testing is reassuring. Our finding of increased vitamin intake in the population testing arm is contrary to two earlier small non‐randomised cohort studies in high‐risk AJ and general population women that showed no short‐term impact on vitamin intake with *BRCA* testing.^9^, ^10^ However, those studies had small sample sizes. Additionally, given the multiple analyses undertaken, our result of increased vitamin intake could be purely a chance finding, as it was not statistically different from the vitamin intake in the FH arm after adjusting for multiple testing. Although we did not specifically assess this, both high prevalence and increased use^21^ of complementary and alternative medicine (CAM) has been reported up to the 1‐year follow‐up following *BRCA* testing in high‐risk individuals. However, the efficacy or benefit of CAM on cancer risk remains unestablished. According to the UK FSA, half of adults (48%) take food supplements (multivitamins being the commonest form) regularly, with another third having taken them in the past.^22^ People take vitamins to maintain/improve their overall health, as a perceived ‘boost’ or to replace nutrients lost due to diet, age or a health condition. Although sales of vitamin supplements have increased over the years, the long‐term health consequences of vitamin consumption are unknown. Consumption in moderation is acceptable, but taking high‐dose supplements is not always effective for disease prevention, and may even be harmful to health.^23^

The decrease in long‐term job‐related physical activity we found is consistent with the reduction in physical activity linked to increasing age in the population. That this was not accompanied by a significant reduction in physical exercise associated with sport or moderate activities such as walking is reassuring. Our findings of lack of health behavioural impact on diet, physical activity, alcohol and smoking is consistent with other reports from high‐risk women.^9^, ^10^ Although a recent report from a population‐based sample suggested that UK women largely anticipated positive engagement with a healthy lifestyle following ovarian/breast cancer risk disclosure, this was not completely borne out in our study.^24^ This could be due to population differences in these studies or the intention–behaviour gap with intent did not completely translate into actions leading to actual lifestyle change.^25^ Our findings are consistent with a recent systematic review which suggests that overall personalised risk information does not lead to a strong effect on health‐related behaviours or sustainable changes to lifestyle.^8^ However, although genetic testing itself may not lead to a significant lifestyle impact, there are some data suggesting that subsequent interventions such as attending a well‐structured patient retreat offering education/information, management updates and support can lead to short‐term positive lifestyle benefits in higher risk women.^26^

Some reports indicate a positive impact on lifestyle following ‘direct‐to‐consumer’ (DTC) genetic testing, in up to 23% participants, on factors such as diet, exercise, vitamin intake, alcohol intake and smoking.^27^, ^28^ However, these were mainly short‐term effects and often related to genetic information with uncertain clinical utility or validity (e.g. SNP profiling). Long‐term data and information on the impact of DTC testing associated with high‐penetrance cancer genes are needed. Some DTC studies show that providing personalised nutrition‐related genetic information can have a positive impact on some dietary components compared with general population‐based dietary advice,^29^ but others show no beneficial impact on physical activity or other lifestyle factors.^30^, ^31^

The reduction in cancer risk perception observed with time following *BRCA* testing is consistent with other reports in the literature from high‐risk studies and cancer risk in non‐carriers in population‐based studies.^19^, ^32^ The lack of difference we found between FH and PS approaches has not been reported before. The lack of impact of population‐based *BRCA* testing on baseline breast screening behaviours is reassuring. Average risk women in the UK undergo 3‐yearly mammograms through the national breast screening programme. Our findings suggest that most women continue to undergo the routine mammograms and although mammogram uptake decreases with age in women over 50 years, this was not different between women undergoing FH‐ or PS‐based *BRCA* testing (Table 1; Table S1). This is congruent with breast screening recommendations for women.^33^

The gender‐based differences in lifestyle related to diet, alcohol and physical activity seen in our study are consistent with differences in health behaviour observed between men and women from the general population.^34^, ^35^, ^36^, ^37^, ^38^, ^39^, ^40^, ^41^, ^42^ Behavioural general population studies have shown that gender differences about beliefs of the importance of healthy eating explain the differences in food choices between men and women. Our results showing that men eat fewer fruits, vegetables and vitamins but consume more meat and alcohol than women, are consistent with the view that part of the reason men report making less healthy food choices is that health is a less important motivation to them in the food domain.^38^, ^39^ Physical activity statistics published by the HSE^42^ and British Heart Foundation are in keeping with our results and show that the mean number of hours per day of moderate or vigorous workplace activity is over twice as high for men as for women (men = 3.8 hours/day, women = 1.7 hours/day).^43^ Similarly, age‐based associations found with diet, alcohol and exercise are akin to normative data from the general population.^39^, ^42^, ^44^, ^45^ A large general population study of drinking behaviour in 35 countries across six continents (Europe, Asia, Africa, Australia, North America, South America) between 1997 and 2007 using a standardised questionnaire revealed a higher prevalence of high‐frequency drinking in older age groups of drinkers and is in keeping with our results.^39^ Our findings support existing literature showing intake of fruit/vegetables/vitamins to be higher but meat consumption lower among older individuals.^45^, ^46^ Our outcomes of higher levels of education being associated with a healthier lifestyle in terms of diet and physical activity are consistent with the reported association of lower education levels with decreased knowledge of genes/cancer risk factors^1^ and the perceived lack of benefit of lifestyle changes on cancer prevention.^24^

Lower levels of education/literacy are linked to lower socio‐economic status, which in turn is associated with a greater sense of powerlessness, belief in chance, poorer social support, limited awareness and lower engagement with screening/prevention. These findings are consistent with the theory of planned behaviour (TPB), where a person's attitudes, subjective norms and perceived behavioural control are key to determining an individual's decision to follow a particular behaviour.^47^ Successful behaviour is further contributed to by actual behavioural control, which refers to the extent to which a person has the skills, resources and other prerequisites (e.g. income, education, social factors) needed to perform the behaviour in question.^48^ Further research is needed to identify reasons for and barriers to a potential intention–behaviour gap^24^ limiting adoption of healthier lifestyles. Research is also needed to quantify levels and types of increased vitamin intake and understand its potential implications.

Most lifestyle outcomes of population testing are comparable to those of FH/criteria‐based testing. These data coupled with other publications from the UK, Israel and Canada ^1^, ^2^, ^3^, ^4^, ^5^, ^7^, ^49^ that show acceptability, feasibility, effectiveness, cost‐effectiveness and lack of detrimental impact on psychological health or quality of life, support the concept of Jewish population‐based *BRCA* testing. The uptake of clinical *BRCA* testing remains limited and only a small proportion of eligible women undergo *BRCA* testing. Correspondingly, only a small proportion of at‐risk *BRCA*‐carriers have been identified^50^, ^51^ The vast majority of *BRCA*‐carriers who can benefit from screening/prevention remain to be identified, missing huge opportunities for precision prevention. Population testing offers the opportunity to maximise carrier identification for primary OC and BC prevention. We^52^, ^53^ and others have argued for changing guidelines in favour of population‐based *BRCA* testing. Israel has recently changed its policy and implemented population *BRCA* testing. It is important that other health systems, including the NHS, follow suit.

## 5 CONCLUSION

Population‐based *BRCA test*ing in the Jewish population does not adversely affect long‐term lifestyle behaviour or routine mammogram screening for average‐risk women. Overall findings from population testing for most outcomes are comparable to those of FH/clinical criteria‐based testing, supporting the concept of population‐based *BRCA* testing in the Jewish population. *BRCA* testing provides the first implementable model for the application of population‐genomics for precision prevention. We call for a change in policy to implement this.

## AUTHOR CONTRIBUTIONS

Conception: RM, IJ. Design & Development: RM, IJ and UM. Questionnaire development: RM, IJ, UM, JW, SS, KL, SG. Data collection: RM, RD, KL. Data analysis: MB, RM. Preparation of tables and figures: RM, MB, FG, MS. Trial management: RM, IJ, UM, KL, RD, JW, SG, LS, HD, YW, CC, IT, UB, AB. Genetic testing: YW. Data collection from Guy’s genetic laboratories: CJ. Initial draft of manuscript: RM, FG, MB, MS. Manuscript writing, review and approval: All authors.

## FUNDING INFORMATION

Following peer review, the study was funded by ‘The Eve Appeal’ charity (grant number GTCV). The funding body had no role in the study design, data collection, analysis, interpretation or writing of the report or decision to submit for publication. The research team was independent of funders. RM is supported by an NHS Innovation Accelerator (NIA) Fellowship for population testing, MB and UM by MRC core funding (MC_UU_00004/01) and UM by the National Institute for Health Research University College London Hospitals Biomedical Research Centre.

The study is supported by researchers at the Barts Cancer Institute Cancer Research UK Centre for Excellence, Queen Mary University of London (C16420/A18066).

The funding body (The Eve Appeal charity) had no role in the study design, data collection, analysis, interpretation or writing of the report or decision to submit for publication. The research team was independent of funders.

## CONFLICT OF INTERESTS

IJ and UM have a financial interest in Abcodia, Ltd., a company formed to develop academic and commercial development of biomarkers for screening and risk prediction. IJ is a member of the board of Abcodia Ltd, a Director of Women's Health Specialists Ltd and received consultancy from Beckton Dickinson. RM declares research funding from The Eve Appeal and Cancer Research UK into population testing and from Barts & the London Charity, Rose Trees Trust and BGCS outside this work; an honorarium for grant review from Israel National Institute for Health Policy Research; and honorarium for advisory board membership or lectures from AstraZeneca/MSD/GSK/EGL. RM is supported by an NHS Innovation Accelerator (NIA) Fellowship for population testing. The other authors declare no conflicts of interest.

## DETAILS OF ETHICS APPROVAL

The GCaPPS study received full ethics approval from the Institute of Child Health/Great Ormond Street Hospital Research Ethics Committee on 8 June 2008 (REC Reference number 08/H0713/44). The study was registered with the International Standard Randomised Controlled Trial Number Register ‐ ISRCTN 73338115 (http://www.controlled‐trials.com/ISRCTN73338115).

All trial volunteers provided written informed consent to participate in the study.

## Supporting information


Table S1

Figure S1.

## Data Availability

The data that support the findings of this study are available from the corresponding author upon reasonable request.
