# Supplementary material for: Randomised trial of population‐based BRCA testing in Ashkenazi Jews: long‐term secondary lifestyle behavioural outcomes
Source: BJOG. 2022 Jul 13;129(12):1970–80. doi: 10.1111/1471-0528.17253 (PMC9796935; doi:10.1111/1471-0528.17253)
Supplement: Supplementary file 1 — Table S1 [file BJO-129-1970-s001.zip › BJO_17253_Revsn_Lifestyle_Suppl_table_BJOG_vR3.docx]

**Table-S1: Generalized Linear Mixed Models for study outcomes**

| **DIET: FRUIT** | **Coef.** | **Std. Err** | **z** | **P>\|z\|** | **[95% CI]** |
| --- | --- | --- | --- | --- | --- |
| Group_PS | 1.113 | 0.697 | 1.60 | 0.110 | -0.253, 2.478 |
| group#year |  |  |  |  |  |
| PS#1 | -0.743 | 0.387 | -1.92 | 0.055 | -1.503, 0.016 |
| PS#2 | -0.336 | 0.398 | -0.84 | 0.399 | -1.116, 0.449 |
| PS#3 | -0.517 | 0.402 | -1.28 | 0.199 | -1.305, 0.271 |
| BRCA status |  |  |  |  |  |
| Negative | 0.733 | 0.881 | 0.83 | 0.405 | -0.993, 2.460 |
| Unknown | 1.399 | 1.082 | 1.29 | 0.196 | -0.722, 3.521 |
| Gender (M) | -1.805 | 0.291 | -6.19 | 0.000 | -2.377, -1.233 |
| Marital Status | 0.305 | 0.350 | 0.87 | 0.384 | -0.381, 0.991 |
| Income |  |  |  |  |  |
| £10,000 - £19,999 | 0.727 | 0.751 | 0.97 | 0.333 | -0.744, 2.199 |
| £20,000 - £29,999 | 0.314 | 0.724 | 0.43 | 0.665 | -1.106, 1.734 |
| £30,000 - £39,999 | 0.755 | 0.686 | 1.10 | 0.271 | -0.589, 2.098 |
| £40,000 - £49,999 | 0.443 | 0.682 | 0.65 | 0.516 | -0.894, 1.779 |
| £50,000 or more | 1.211 | 0.626 | 1.93 | 0.053 | -0.016, 2.437 |
| Education | 0.879 | 0.292 | 3.01 | 0.003 | 0.307, 1.451 |
| FH (Low-risk) | -0.507 | 0.593 | -0.85 | 0.393 | -1.669, 0.655 |
| Age | 0.064 | 0.010 | 6.11 | <0.0001 | 0.043, 0.084 |
|  |  |  |  |  |  |
| **DIET: RED MEAT** | **Coef.** | **Std. Err** | **z** | **P>\|z\|** | **[95% CI]** |
| Group_PS | -1.559 | 0.811 | -1.92 | 0.054 | -3.148, 0.030 |
| group#year |  |  |  |  |  |
| PS#1 | -0.257 | 0.319 | -0.810 | 0.419 | -0.882, 0.368 |
| PS#2 | -0.003 | 0.329 | -0.01 | 0.993 | -0.647, 0.642 |
| PS#3 | -0.013 | 0.334 | -0.04 | 0.967 | -0.668, 0.641 |
| BRCA status |  |  |  |  |  |
| Negative | -2.115 | 1.11 | -1.91 | 0.057 | -4.29, 0.061 |
| Unknown | -2.971 | 1.367 | -2.17 | 0.030 | -5.65, -0.292 |
| Gender (M) | 1.844 | 0.354 | 5.2 | <0.0001 | 1.149, 2.538 |
| Marital Status | 0.119 | 0.430 | 0.28 | 0.782 | -0.724, 0.962 |
| Income |  |  |  |  |  |
| £10,000 - £19,999 | -0.416 | 1.013 | -0.41 | 0.681 | -2.402, 1.570 |
| £20,000 - £29,999 | 0.049 | 0.952 | 0.05 | 0.959 | -1.817, 1.916 |
| £30,000 - £39,999 | -1.556 | 0.943 | -1.65 | 0.099 | -3.404, 0.292 |
| £40,000 - £49,999 | 0.130 | 0.928 | 0.14 | 0.888 | -1.688, 1.949 |
| £50,000 or more | -0.110 | 0.852 | -0.13 | 0.897 | -1.779, 1.559 |
| Education | -0.137 | 0.353 | -0.39 | 0.699 | -0.829, 0.556 |
| FH (Low-risk) | 1.02 | 0.681 | 1.50 | 0.134 | -0.314, 2.355 |
| Age | -0.024 | 0.012 | -2.03 | 0.043 | -0.047, -0.001 |
|  |  |  |  |  |  |
| **DIET: VEGETABLE** | **Coef.** | **Std. Err** | **z** | **P>\|z\|** | **[95% CI]** |
| Group_PS | 0.932 | 0.702 | 1.33 | 0.184 | -0.443, 2.308 |
| group#year |  |  |  |  |  |
| PS#1 | 0.002 | 0.386 | 0.01 | 0.996 | -0.754, 0.758 |
| PS#2 | -0.373 | 0.393 | -0.95 | 0.343 | -1.143, 0.397 |
| PS#3 | -0.174 | 0.400 | -0.43 | 0.665 | -0.959, 0.612 |
| BRCA status |  |  |  |  |  |
| Negative | 0.354 | 0.918 | 0.39 | 0.700 | -1.446, 2.154 |
| Unknown | 1.154 | 1.095 | 1.05 | 0.292 | =0.991, 3.30 |
| Gender (M) | -2.262 | 0.298 | -7.57 | <0.0001 | -2.848, -1.677 |
| Marital Status | 0.649 | 0.348 | 1.86 | 0.062 | -0.033, 1.332 |
| Income |  |  |  |  |  |
| £10,000 - £19,999 | 0.367 | 0.769 | 0.48 | 0.633 | -1.14, 1.875 |
| £20,000 - £29,999 | 0.995 | 0.738 | -1.35 | 0.177 | -2.44, 0.451 |
| £30,000 - £39,999 | 0.001 | 0.702 | 0.00 | 1 | -1.377, 1.377 |
| £40,000 - £49,999 | -0.062 | 0.714 | -0.09 | 0.931 | -1.462, 1.338 |
| £50,000 or more | 0.678 | 0.652 | 1.04 | 0.298 | -0.599, 1.956 |
| Education | 1.074 | 0.292 | 3.68 | <0.0001 | 0.502, 1.647 |
| FH (Low-risk) | -0.446 | 0.573 | -0.78 | 0.436 | -1.568, 0.677 |
| Age | 0.020 | 0.010 | 2.00 | 0.046 | 0.001, 0.040 |
|  |  |  |  |  |  |
| **VITAMINS** | **Coef.** | **Std. Err** | **z** | **P>\|z\|** | **[95% CI]** |
| Group_PS | -1.192 | 0.637 | -1.87 | 0.061 | -2.44 - 0.560 |
| group#year |  |  |  |  |  |
| PS#1 | 0.457 | 0.324 | 1.41 | 0.158 | -0.178, 1.093 |
| PS#2 | 0.639 | 0.331 | 1.93 | 0.054 | -0.01, 1.29 |
| PS#3 | 0.979 | 0.340 | 2.88 | 0.004 | 0.312, 1.645 |
| BRCA status |  |  |  |  |  |
| Negative | -0.149 | 0.885 | -0.17 | 0.866 | -1.884, 1.585 |
| Unknown | -0.814 | 1.042 | -0.78 | 0.435 | -2.857, 1.229 |
| Gender (M) | -1.597 | 0.273 | -5.85 | <0.0001 | -2.132, -1.062 |
| Marital Status | -0.505 | 0.321 | -1.57 | 0.116 | -1.134, 0.125 |
| Income |  |  |  |  |  |
| £10,000 - £19,999 | 0.901 | 0.715 | 1.26 | 0.208 | -0.502, 2.303 |
| £20,000 - £29,999 | 1.032 | 0.694 | 1.49 | 0.137 | -0.328, 2.392 |
| £30,000 - £39,999 | 0.746 | 0.694 | 1.13 | 0.258 | -0.546, 2.037 |
| £40,000 - £49,999 | 0.346 | 0.659 | 0.52 | 0.604 | -0.959, 1.65 |
| £50,000 or more | -0.041 | 0.608 | -0.07 | 0.946 | -1.234-1.151 |
| Education | -0.076 | 0.262 | -0.29 | 0.771 | -0.590, 0.438 |
| FH (Low-risk) | 0.316 | 0.511 | 0.62 | 0.536 | -0.684, 1.317 |
| Age | 0.038 | 0.009 | 4.10 | <0.0001 | 0.020, 0.056 |
|  |  |  |  |  |  |
| **ALCOHOL: QUANTITY** | **Coef.** | **Std. Err** | **z** | **P>\|z\|** | **[95% CI]** |
| Group_PS | -0.552 | 0.698 | -0.79 | 0.429 | -1.921, 0.816 |
| group#year |  |  |  |  |  |
| PS#1 | -0.050 | 0.298 | -0.170 | 0.868 | -0.633, 0.534 |
| PS#2 | 0.560 | 0.303 | 1.850 | 0.064 | -0.033, 1.153 |
| PS#3 | -0.200 | 0.307 | -0.650 | 0.514 | -0.802, 0.402 |
| BRCA status |  |  |  |  |  |
| Negative | -1.345 | 0.976 | -1.38 | 0.168 | -3.257, 0.567 |
| Unknown | -2.125 | 1.159 | -1.83 | 0.067 | -4.397, 0.147 |
| Gender (M) | 2.483 | 0.305 | 8.14 | <0.0001 | 1.884, 3.080 |
| Marital Status | -1.286 | 0.358 | -3.59 | <0.0001 | -1.988, -0.584 |
| Income |  |  |  |  |  |
| £10,000 - £19,999 | -0.094 | 0.794 | -0.12 | 0.906 | -1.651, 1.463 |
| £20,000 - £29,999 | 0.067 | 0.774 | 0.09 | 0.931 | -1.450, 1.584 |
| £30,000 - £39,999 | 0.912 | 0.735 | 1.24 | 0.214 | -0.528, 2.353 |
| £40,000 - £49,999 | 1.499 | 0.745 | 2.00 | 0.045 | 0.032, 2.966 |
| £50,000 or more | 2.164 | 0.683 | 3.17 | 0.002 | 0.826, 3.502 |
| Education | 0.189 | 0.293 | 0.65 | 0.518 | -0.385, 0.765 |
| FH (Low-risk) | 1.01 | 0.562 | 1.80 | 0.072 | -0.091, 2.113 |
| Age | 0.037 | 0.010 | 3.62 | <0.0001 | 0.017, 0.057 |
|  |  |  |  |  |  |
| **ALCOHOL: FREQUENCY** | **Coef.** | **Std. Err** | **z** | **P>\|z\|** | **[95% CI]** |
| Group_PS | -0.256 | 0.870 | -0.29 | 0.768 | -1.96, 1.449 |
| group#year |  |  |  |  |  |
| PS#1 | 0.575 | 0.249 | 2.300 | 0.021 | 0.085, 1.065 |
| PS#2 | 0.519 | 0.256 | 2.03 | 0.042 | 0.018, 1.020 |
| PS#3 | 0.328 | 0.261 | 1.25 | 0.210 | -0.184, 0.841 |
| BRCA status |  |  |  |  |  |
| Negative | -1.263 | 1.197 | -1.06 | 0.291 | -3.61, 1.083 |
| Unknown | -2.395 | 1.411 | -1.70 | 0.090 | -5.161, 0.371 |
| Gender (M) | 3.064 | 0.390 | 7.86 | <0.0001 | 2.30, 3.83 |
| Marital Status | -1.349 | 0.465 | -2.90 | 0.004 | -2.261, -0.437 |
| Income |  |  |  |  |  |
| £10,000 - £19,999 | 1.325 | 1.00 | 1.33 | 0.185 | -0.635, 3.286 |
| £20,000 - £29,999 | 1.167 | 0.989 | 1.18 | 0.238 | -0.771, 3.104 |
| £30,000 - £39,999 | 3.233 | 0.946 | 3.42 | 0.001 | 1.379, 5.087 |
| £40,000 - £49,999 | 3.026 | 0.951 | 3.18 | 0.001 | 1.163, 4.89 |
| £50,000 or more | 4.838 | 0.872 | 5.55 | <0.0001 | 3.129, 6.547 |
| Education | 0.759 | 0.383 | 1.98 | 0.047 | 0.009, 1.509 |
| FH (Low-risk) | 1.262 | 0.718 | 1.76 | 0.079 | -0.145, 2.669 |
| Age | 0.066 | 0.013 | 5.02 | <0.0001 | 0.040, 0.091 |
|  |  |  |  |  |  |
| **PHYSICAL ACTIVITY: EXERCISE** | **Coef.** | **Std. Err** | **z** | **P>\|z\|** | **[95% CI]** |
| Group_PS | 1.127 | 0.553 | 2.04 | 0.042 | 0.042, 2.21 |
| group#year |  |  |  |  |  |
| PS#1 | -0.306 | 0.325 | -0.94 | 0.346 | -0.942, 0.330 |
| PS#2 | 0.306 | 0.339 | 0.90 | 0.367 | -0.358, 0.970 |
| PS#3 | -0.215 | 0.346 | -0.62 | 0.534 | -0.893, 0.463 |
| BRCA status |  |  |  |  |  |
| Negative | -0.670 | 0.777 | -0.86 | 0.389 | -2.193, 0.854 |
| Unknown | 0.125 | 0.919 | 0.14 | 0.891 | -1.676, 1.927 |
| Gender (M) | -0.261 | 0.228 | -1.14 | 0.253 | -0.709, 0.186 |
| Marital Status | -0.053 | 0.276 | -0.19 | 0.849 | -0.593, 0.488 |
| Income |  |  |  |  |  |
| £10,000 - £19,999 | 0.349 | 0.585 | 0.60 | 0.550 | -0.797, 1.496 |
| £20,000 - £29,999 | 0.922 | 0.577 | 1.60 | 0.110 | -0.209, 2.053 |
| £30,000 - £39,999 | 1.242 | 0.549 | 2.26 | 0.024 | 0.167, 2.318 |
| £40,000 - £49,999 | 0.946 | 0.550 | 1.72 | 0.086 | -0.133, 2.025 |
| £50,000 or more | 1.494 | 0.502 | 2.98 | 0.003 | 0.510, 2.480 |
| Education | 0.055 | 0.228 | 0.240 | 0.810 | -0.392, 0.502 |
| FH (Low-risk) | 0.328 | 0.449 | -0.73 | 0.464 | -1.208, 0.552 |
| Age | 0.005 | 0.008 | 0.68 | 0.496 | -0.10, 0.02 |
|  |  |  |  |  |  |
| **PHYSICAL ACTIVITY: WALKING/ MODERATE ACTIVITY** | **Coef.** | **Std. Err** | **z** | **P>\|z\|** | **[95% CI]** |
| Group_PS | 0.623 | 0.653 | 0.95 | 0.341 | -0.658, 1.903 |
| group#year |  |  |  |  |  |
| PS#1 | 0.267 | 0.347 | 0.77 | 0.442 | -0.414, 0.948 |
| PS#2 | 0.438 | 0.359 | 1.22 | 0.223 | -0.266, 1.141 |
| PS#3 | 0.435 | 0.366 | 1.19 | 0.235 | -0.283, 1.153 |
| BRCA status |  |  |  |  |  |
| Negative | -0.205 | 0.88 | -0.23 | 0.816 | -1.93, 1.52 |
| Unknown | 0.775 | 1.047 | 0.74 | 0.459 | -1.28, 2.83 |
| Gender (M) | 0.225 | 0.267 | 0.84 | 0.398 | -0.298, 0.750 |
| Marital Status | -0.306 | 0.327 | -0.93 | 0.350 | -0.948, 0.336 |
| Income |  |  |  |  |  |
| £10,000 - £19,999 | 0.769 | 0.725 | 1.06 | 0.289 | -0.652, 2.191 |
| £20,000 - £29,999 | -0.469 | 0.699 | -0.67 | 0.502 | -1.838, 0.900 |
| £30,000 - £39,999 | 0.810 | 0.665 | 1.22 | 0.223 | -0.493, 2.114 |
| £40,000 - £49,999 | -0.152 | 0.666 | -0.23 | 0.819 | -1.458, 1.153 |
| £50,000 or more | 0.262 | 0.609 | 0.43 | 0.667 | -0.931, 1.456 |
| Education | -0.061 | 0.268 | -0.23 | 0.821 | -0.586, 0.465 |
| FH (Low-risk) | -0.868 | 0.528 | -1.64 | 0.1 | -1.9, 0.167 |
| Age | 0.017 | 0.009 | 1.80 | 0.071 | -0.001, 0.034 |
|  |  |  |  |  |  |
| **PHYSICAL ACTIVITY: JOB** | **Coef.** | **Std. Err** | **z** | **P>\|z\|** | **[95% CI]** |
| Group_PS | -0.653 | 0.661 | -0.99 | 0.324 | -1.948, 0.643 |
| group#year |  |  |  |  |  |
| PS#1 | -0.014 | 0.281 | -0.05 | 0.960 | -0.565, 0.537 |
| PS#2 | 0.057 | 0.294 | 0.19 | 0.846 | -0.519, 0.633 |
| PS#3 | -0.043 | 0.302 | -0.14 | 0.888 | -0.635, 0.550 |
| BRCA status |  |  |  |  |  |
| Negative | -2.435 | 0.965 | -2.52 | 0.012 | -4.326, -0.543 |
| Unknown | -3.262 | 1.131 | -2.88 | 0.004 | -5.48, -1.044 |
| Gender (M) | 0.709 | 0.275 | 2.57 | 0.010 | 0.169, 1.249 |
| Marital Status | -0.697 | 0.341 | -2.04 | 0.041 | -1.365, -0.027 |
| Income |  |  |  |  |  |
| £10,000 - £19,999 | -0.575 | 0.832 | -0.69 | 0.489 | -2.206, 1.056 |
| £20,000 - £29,999 | 0.506 | 0.792 | 0.64 | 0.523 | -1.048, 2.060 |
| £30,000 - £39,999 | 0.790 | 0.754 | 1.05 | 0.295 | -0.687, 2.267 |
| £40,000 - £49,999 | 0.883 | 0.745 | 1.19 | 0.236 | -0.576, 2.343 |
| £50,000 or more | 1.390 | 0.683 | 2.03 | 0.042 | 0.05, 2.73 |
| Education | 0.862 | 0.285 | 3.03 | 0.002 | 0.304, 1.419 |
| FH (Low-risk) | 0.715 | 0.538 | 1.33 | 0.184 | -0.339, 1.77 |
| Age | 0.003 | 0.010 | 0.34 | 0.732 | -0.017, 0.023 |
|  |  |  |  |  |  |
| ***SMOKING: FREQUENCY** | **Coef.** | **Std. Err** | **z** | **P>\|z\|** | **[95% CI]** |
| Group_PS | 0.015 | 0.915 | 0.020 | 0.987 | -1.779-1.809 |
| group#year |  |  |  |  |  |
| PS#1 | 0.004 | 0.922 | 0.000 | 0.997 | -1.803-1.811 |
| PS#2 | -1.003 | 0.930 | -1.080 | 0.281 | -2.826-0.820 |
| PS#3 | -1.311 | 0.989 | -1.080 | 0.281 | -2.826-0.820 |
| Gender (M) | -0.148 | 0.868 | -0.170 | 0.865 | -1.849-1.553 |
| Marital Status | -0.698 | 0.915 | -0.760 | 0.445 | -2.491-1.095 |
| Income |  |  |  |  |  |
| £10,000 - £19,999 | -1.262 | 1.712 | -0.740 | 0.461 | -4.617-2.093 |
| £20,000 - £29,999 | -22.477 | 5.205 | -4.320 | 0.000 | -32.679-12.274 |
| £30,000 - £39,999 | -1.647 | 1.522 | -1.080 | 0.279 | -4.629-1.335 |
| £40,000 - £49,999 | -1.186 | 1.486 | -0.800 | 0.425 | -4.098-1.726 |
| £50,000 or more | -1.733 | 1.345 | -1.290 | 0.198 | -4.370-0.904 |
| Education | -0.703 | 0.855 | -0.820 | 0.411 | -2.380-0.973 |
| FH (Low-risk) | 0.110 | 1.364 | 0.080 | 0.936 | -2.563-2.783 |
| Age | -0.022 | 0.027 | -0.820 | 0.412 | -0.075-0.031 |
|  |  |  |  |  |  |
| ***SMOKING: STOPPING** | **Coef.** | **Std. Err** | **z** | **P>\|z\|** | **[95% CI]** |
| Group_PS | 1.110 | 0.840 | 1.320 | 0.187 | -0.538-2.757 |
| group#year |  |  |  |  |  |
| PS#1 | -0.138 | 1.168 | -0.120 | 0.906 | -2.428-2.152 |
| PS#2 | -1.551 | 1.196 | -1.300 | 0.195 | -3.896-0.794 |
| PS#3 | 0.672 | 1.227 | 0.550 | 0.584 | -1.733-3.076 |
| Gender (M) | 0.725 | 0.646 | 1.120 | 0.262 | -0.541-1.991 |
| Marital Status | -1.075 | 0.736 | -1.460 | 0.144 | -2.517-0.367 |
| Income |  |  |  |  |  |
| £10,000 - £19,999 | 0.271 | 1.503 | 0.180 | 0.857 | -2.675-3.217 |
| £20,000 - £29,999 | -1.043 | 1.744 | -0.600 | 0.550 | -4.462-2.376 |
| £30,000 - £39,999 | 1.184 | 1.471 | 0.810 | 0.421 | -1.699-4.068 |
| £40,000 - £49,999 | -1.062 | 1.391 | -0.760 | 0.445 | -3.789-1.665 |
| £50,000 or more | -0.354 | 1.225 | -0.290 | 0.773 | -2.755-2.047 |
| Education | -0.749 | 0.625 | -1.200 | 0.231 | -1.974-0.476 |
| FH (Low-risk) | 1.215 | 1.008 | 1.210 | 0.228 | -0.761-3.191 |
| Age | -0.007 | 0.024 | -0.310 | 0.757 | -0.054-0.039 |
|  |  |  |  |  |  |
| **CANCER RISK PERCEPTION** | **Coef.** | **Std. Err** | **z** | **P>\|z\|** | **[95% CI]** |
| Group_PS | 0.922 | 0.564 | 1.63 | 0.102 | -1.84, 2.028 |
| group#year |  |  |  |  |  |
| PS#1 | -0.394 | 0.255 | -1.54 | 0.123 | -0.894, 0.106 |
| PS#2 | -0.343 | 0.260 | -1.32 | 0.188 | -0.853, 0.167 |
| PS#3 | -0.436 | 0.266 | -1.64 | 0.101 | -0.956, 0.084 |
| BRCA status |  |  |  |  |  |
| Negative | -3.227 | 0.797 | -4.-05 | <0.0001 | -4.79, -1.67 |
| Unknown | -2.308 | 0.941 | -2.45 | 0.014 | -4,153, -0.463 |
| Gender (M) | -0.607 | 0.238 | -2.55 | 0.011 | -1.075, -0.139 |
| Marital Status | -0.372 | 0.289 | -1.29 | 0.198 | -0.939, 0.194 |
| Income |  |  |  |  |  |
| £10,000 - £19,999 | -0.080 | 0.641 | -0.12 | 0.901 | -1.336, 1.176 |
| £20,000 - £29,999 | -0.501 | 0.626 | -0.80 | 0.424 | -1.728, 0.726 |
| £30,000 - £39,999 | -0.329 | 0.594 | -0.55 | 0.579 | -1.493, 0.835 |
| £40,000 - £49,999 | -0.266 | 0.601 | -0.44 | 0.657 | -1.444, 0.910 |
| £50,000 or more | -0.174 | 0.545 | -0.32 | 0.749 | -1.242, 0.892 |
| Education | 0.218 | 0.239 | 0.91 | 0.362 | -0.251, 0.687 |
| FH (Low-risk) | -2.69 | 0.461 | -5.84 | <0.0001 | -3.594, -1.787 |
| Age | -0.040 | 0.008 | -4.78 | <0.0001 | -0.056, -0.023 |
|  |  |  |  |  |  |
| **MAMMOGRAM** | **Coef.** | **Std. Err** | **z** | **P>\|z\|** | **[95% CI]** |
| Group_PS | 0.055 | 0.538 | 0.10 | 0.918 | -0.999, 1.110 |
| group#year |  |  |  |  |  |
| PS#1 | -0.195 | 0.381 | -0.51 | 0.608 | -0.942, 0.551 |
| PS#2 | -0.104 | 0.377 | -0.28 | 0.782 | -0.843, 0.634 |
| PS#3 | -0.350 | 0.379 | -0.92 | 0.357 | -1.094, 0.394 |
| BRCA status |  |  |  |  |  |
| Negative | -0.362 | 0.811 | -0.45 | 0.655 | -1.952, 1.228 |
| Unknown | -0.536 | 0.906 | -0.59 | 0.554 | -2.311, 1.239 |
| Gender (M) |  |  |  |  |  |
| Marital Status | 0.129 | 0.233 | 0.55 | 0.581 | -0.329, 0.587 |
| Income |  |  |  |  |  |
| £10,000 - £19,999 | -0.024 | 0.461 | -0.05 | 0.958 | -0.928, 0.880 |
| £20,000 - £29,999 | 0.988 | 0.461 | 0.214 | 0.032 | 0.085, 1.892 |
| £30,000 - £39,999 | 0.927 | 0.442 | 0.210 | 0.036 | -0.061, 1.793 |
| £40,000 - £49,999 | 0.696 | 0.472 | 1.47 | 0.141 | -0.230, 1.623 |
| £50,000 or more | 0.720 | 0.424 | 1.70 | 0.090 | -0.112, 1.552 |
| Education | 0.003 | 0.193 | 0.01 | 0.989 | -0.376, 0.381 |
| FH (Low-risk) | -0.414 | 0.377 | -1.1 | 0.273 | -1.153, 0.325 |
| Age | -0.053 | 0.013 | -4.04 | <0.0001 | -0.078, -0.027 |

PS- population screening, FH- family history, M- male, Coef- coefficient, Std. Err- Standard error, CI- confidence interval

Group-term: ‘population-screening’ or ‘family-history’ (reference category)

Gender: Men versus Women (reference category),

Marital status: married or cohabiting versus widowed, divorced or single (reference category)

Income <£10,000 (reference category), £10,000 to <£20,000, £20,000 to <£30,000, £30,000 to <£40,000, £40,000 to <£50,000 and >£50,000),

Education: degree-level or above versus no formal qualification/GCSE/O-level/CSE/NVQ1/NVQ2/A-level education (reference category),

Family-history: low-risk versus high-risk (reference category)

BRCA Status: BRCA positive (reference category), BRCA negative, BRCA Unknown

*Smoking Frequency and Smoking Stopping models could not be adjusted for BRCA status as all BRCA carriers were non-smokers.

**Supplementary** **Figure S1- Consort flow chart for the study**

FH – Family history, PS- population screening, Pos- positive, Neg- negative
