# Supplementary figures and images for: Randomised trial of population‐based BRCA testing in Ashkenazi Jews: long‐term secondary lifestyle behavioural outcomes
Source: BJOG. 2022 Jul 13;129(12):1970–80. doi: 10.1111/1471-0528.17253 (PMC9796935; doi:10.1111/1471-0528.17253)

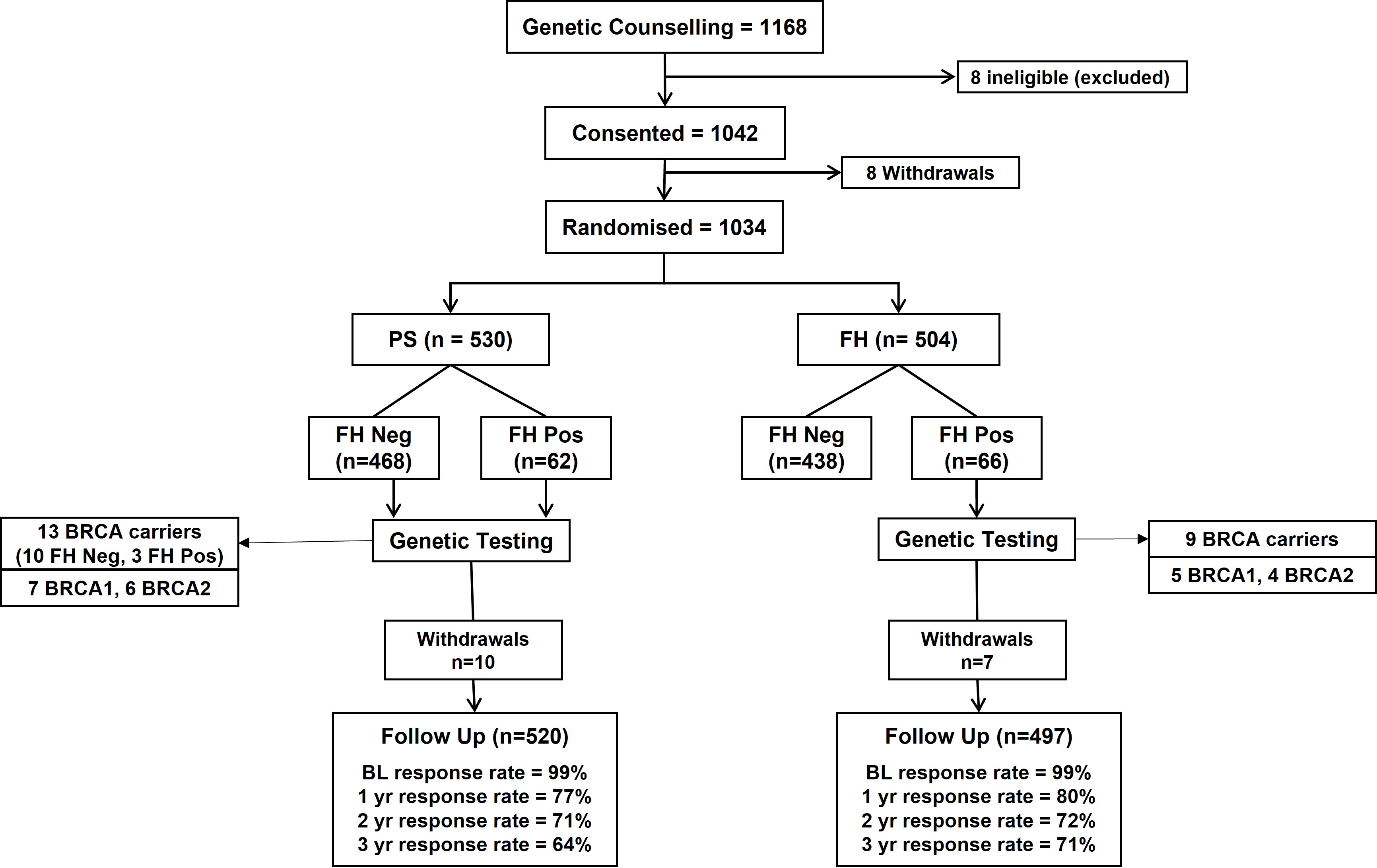

Supplement: Supplementary file 2 — Figure S1. [file BJO-129-1970-s002.jpg]
